# Female genital image: is there a relationship with body image?

**DOI:** 10.4274/tjod.galenos.2019.49799

**Published:** 2019-07-03

**Authors:** Tamara Barbara Silva Gomes, Cristina Aires Brasil, Ana Paula Pitia Barreto, Roseny Santos Ferreira, Bary Berghmans, Patricia Lordelo

**Affiliations:** 1 Bahiana School of Medicine and Public Health, Center for Attention to the Pelvic Floor, Course of Physiotherapy, Bahia, Brazil; 2 Maastricht University Medical Centre, Pelvic Care Center Maastricht, Department of Epidemiology at Maastricht University, Department of Urology, Maastricht, Netherlands

**Keywords:** Body image, female genitalia, women, genital (self-)image

## Abstract

**Objective:**

Dissatisfaction with body image may extend to the genital region, and the most dissatisfied with their bodies are women. To analyze the relation between body image and genital image in female, and to verify demographic and/or clinical factors related to body image and genital image.

**Materials and Methods:**

This is a cross-sectional study in 421 women. The Body Shape Questionnaire-34 (BSQ-34) was used to evaluate body image perception; scores ≤110 indicate no dissatisfaction. Also, the female genital self-image scale-7 (FGSIS-7) was used to evaluate genital self-image; scores range between 7 and 28, with higher values considered to indicate a more positive genital self-image. The relation between body image and genital image was determined using the Pearson Correlation test, as well as the relation of these with body mass index (BMI) and age. The relation between these data and genital image was determined by using the ANOVA test or the independent t-test (statistical difference was accepted as p<0.05). In order to verify predictors of dissatisfaction with body image, variables with p<0.10 were inserted into the logistic regression model and checked if they remained significant (p<0.05).

**Results:**

Three hundred eighty-nine women were analyzed. The mean age was 34.7±10.2 years. The mean BMI was 24.1±3.6 kg/m², 49% were single, and the mean BSQ-34 and FGSIS scores were 83.2±30.8 and 23.8±3.4, respectively. The correlation (r=-0.24) was found between body image and genital image (p<0.001). A total of 315 women indicated to be satisfied with their body and presented an FGSIS-7 score of 24±3.3. Participants who were dissatisfied with their body had an average FGSIS-7 score of 22.6±3.3.

**Conclusion:**

Genital image, age, and BMI influence body image. Change in the perception of body image seems to have low correlation with genital self-image in women.

**PRECIS:** The dissatisfaction with body image is associated with poorer genital self-image.

## Introduction

The dissatisfaction with body self-image in the Brazilian adult population is around 60 to 87%. In a general way, women are the most dissatisfied with their bodies, and being overweight was the main reason for body dissatisfaction^(1,2,3,4)^. Dissatisfaction in relation to one’s own body may affect one’s health in various ways, for example in a psychosocial and/or nutritional way, or in behavior towards the practice of physical activities, sexual health, as well as the search for esthetic procedures^(1,5,6)^.

Women’s perception of body image may extend to different parts of the body, including their intimate region^(7,8)^. Attention to the external genitalia has grown in parallel with pubic hair depilation tendencies, which results in a greater exposure of the sexual organs^(9,10)^. As genitalia are more exposed, the degree of satisfaction with external genital organs can be influenced, as well as women’s sexual experiences^(11,12)^.

In Brazil, body exposure and body image are highly valued by men and women due to strong socio-cultural influences^(13)^. However, it is still unknown if there is a relation between the cult of the body and female genital image. What could be observed is that Brazilian women, who undergo many esthetic body procedures, also perform innumerable surgeries in the genital region. In the last survey conducted by the International Society of Aesthetic Plastic Surgeons (ISAPS) in 2014, it was possible to observe that Brazil was the country where the most genital esthetic surgeries were performed. Moreover, this surgery was one of the most performed in the country^(14)^. Just as in Turkey, the main motivator for labiaplasty was found as improvement in genital appearance integrated with their esthetic and sexual demands^(15)^.

In the literature, we found studies that verified the relation between body-image and female genital image in a university population, and in women with dyspareunia^(8,16)^. However, the relation between the level of satisfaction of Brazilian women with genital self-image is not well defined. The objective of this study was to analyze the relation between body and genital image, as well as to verify demographic and/or clinical factors related to them.

## Materials and Methods

This is a cross-sectional study. Adult women recruited at health promotion events conducted by the Pelvic Floor Care Center in the cities of Salvador-Bahia in Brazil were invited to participate in the survey from February to June 2015. Seventy women aged between 18 and 60 years who were members of health clubs were included in the study. Pregnant volunteers and those who did not complete the submitted assessment instruments were excluded from the research.

### Procedures of data collection

The volunteers were informed about the study objectives by previously trained researchers. After expressing an interest in participating, they were directed to a separate place and given instructions to complete the self-administered questionnaires individually. The research was conducted using evaluation instruments to collect socio-demographic and clinical information, the Body Shape Questionnaire (BSQ-34) and the female genital self-image scale (FGSIS-7).

### Evaluation tools

### Body image

The BSQ-34 is an instrument that was validated for use in the Portuguese language by Di Pietro & Silveira, in 2009. It consists of 34 questions and was developed to measure concerns of body image and weight over the previous four weeks. The tool provides an evaluation on body image dissatisfaction in clinical and research environments^(17)^.

The questions refer to the degree of concerns with body shape and weight, self-depreciation related to clinical appearance, and behavioral modifications. The answers have scores ranging from one to six, representing the options: never, rarely, sometimes, often, very often, and always, respectively. The final score can range from 34 to 204 points, with a value less than or equal to 110 indicating no concerns, a value greater than 110 and less than or equal to 138 indicating mild concern, a score greater than 138 and less than or equal to 167 corresponding to a moderate concern, and a score greater than 167 is indicative of serious bodily concern^(18)^.

### Genital image

The assessment of satisfaction and women’s beliefs regarding their own genitalia was performed by using the FGSIS-7. This is a reliable questionnaire consisting of seven questions with a four-point answer scale in descending order (i totally agree, agree, disagree, strongly disagree). The seven items in the questionnaire include smell and taste, appearance, sexual function, shame, and pride. The total score can range from 7 to 28 points, there is no cut-off point, and higher scores indicate a more positive self-image of the genitalia^(12)^.

Herbenick et al.,^(12)^ recommended that the absence of a response justified the exclusion of the analysis from the scale. The FGSIS-7 has been translated and validated for some Western and Eastern countries and is considered a reliable measurement tool^(19,20,21,22)^. To date, the instrument has not been validated in Brazil, but it is in the process of validation. The authors translated the scale because no other validated scale or questionnaire was found to assess genital self-image.

### Statistics

The correlation variables corresponded to the BSQ-34 questionnaires and to FGSIS. The correlation variables corresponding to the BSQ-34 questionnaires provided a numeric variable, and scores less than or equal to 110 were considered to indicate body image satisfaction because of an absence of concerns related to the body^(18)^. FGSIS-7 provides a numerical variable in which higher values represent a more positive genital self-image^(19)^.

If the body mass index (BMI) is less than 18.5 kg/m^2^, it falls into the underweight range. If the BMI is 18.5 to <25 kg/m^2^, it falls within the normal range. If the BMI is 25.0 to <30 kg/m^2^, it falls within the overweight range. If the BMI is 30.0 kg/m^2^ or higher, it is regarded as obesity.

In order to derive the sample size of this research, the Winpepi calculator was used, through the ETCETERA command (miscellaneous procedures) in order to obtain a sample size through the correlation coefficient. The parameters were: correlation coefficient 0.2^(16,19)^ and a power of 80% with a significance of 5%, obtaining n of 194 participants, adding 10% of possible losses, and 214 individuals in the aggregate.

### Statistical Analysis

In order to prepare the database and descriptive analysis, the Statistical Package for the Social Sciences (SPSS Inc., Chicago, IL, USA), version 14.0 for Windows was used. The normality of the numeric variables was examined through descriptive statistics, graphical analysis, and the Kolmogorov-Smirnov test.

The correlation between the scores of BSQ-34 and FGSIS-7 questionnaires was evaluated by means of Pearson’s correlation, as well as the correlation with age and BMI. For the purpose of comparison of categorical variables (BSQ-34 categorical vs. schooling, marital status, medication-hormone use, type of delivery and previous pelvic surgery), the chi-square test was used. In order to compare the means between categorical BSQ-34 with FGSIS-7, age and BMI, the independent t-test was used.

The multiple logistic regression model was used to evaluate the possibility to predict each independent variable in case of a change in body image using the BSQ-34. The multiple logistic regression model was used to evaluate the predictive ability of each independent variable in a change in outcome regarding body image using BSQ-34. After the univariate analysis, the variables were inserted into the logistic model if p<0.10, and they were kept in the model if they remained significant (p<0.05). The manual procedure for insertion and withdrawal of the variables was adopted. The power of discrimination of the model was determined using the area under the receiver operating characteristics (ROC) curve, represented by the C statistic, allowing to define the capacity to discriminate those with and without body alterations. The calibration of the model was verified using the Hosmer and Lemeshow test.

This study was approved by the Research Ethics Committee of the Bahia School of Medicine and Public Health, where it received the following CAAE number: 14425813.9.0000.5544. All patients signed the Free and informed consent form.

## Results

The sample comprised 387 women. Initially, 423 volunteers participated in this study, with a loss of 35 participants because they did not complete the BSQ-34 questionnaire and one for not completing the FGSIS-7. Table 1 describes the socio-demographic and clinical data of the sample.

A negative and weak correlation was identified between the BSQ-34 and FGSIS-7 questionnaires, with a correlation coefficient of -0.240 (p<0.001), whereby the higher the body satisfaction the better the perception of the genital self-image.

The women in this study were divided into two groups: those who were satisfied and dissatisfied with their body image. When comparing the FGSIS-7 score between these two groups, it was observed that women who were dissatisfied with their body presented a worse genital image, derived from a lower score in the evaluation instrument (p=0.002). In the comparison of the means of clinical and socio-demographic data, it was verified that women who were dissatisfied with their body presented a higher BMI and lower age (p<0.05). The data are presented in Table 2.

**Figure 1:**
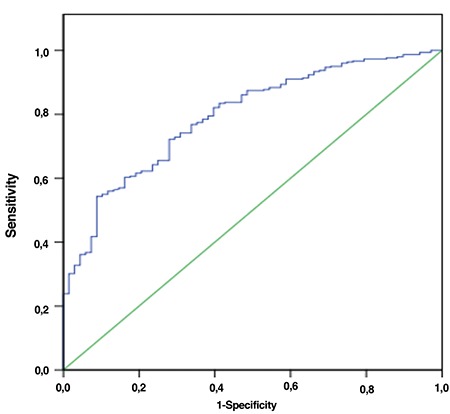
Area under the ROC curve of the final model (BMI, age and FGSIS) for body image ROC: Receiver operating characteristic, BMI: Body mass index, FGSIS: Female genital self-image scale

Analysis of predictors of body image with genital image and socio-demographic and clinical data showed that female genital self-imaging, age, and BMI influenced body image (p<0.05) (Table 3). The ROC curve was used to represent the relationship between age, BMI, and FGSIS with body image. Through this final model, an ROC curve was developed that obtained an area of 0.79 (95% CI: 0.74-0.85), with p<0.001, as described in Figure 1.

## Discussion

To date, studies that evaluate the relationship between body and genital image are scarce, and nonexistent in the Brazilian female population. In the present study, a correlation was verified between female body and genital images. Participants dissatisfied with their body image, due to concerns about their body, were more dissatisfied with their genital self-image. By examining the satisfaction between body and genital image in female university students, research revealed that women who were more satisfied with their genitalia were more satisfied with their body^(7,16,19)^.

Pazmany et al.,^(8)^ described the relation between body and genital image of a sample that was divided into two groups: women with self-reported dyspareunia and a control group with women who did not report pain during or after sexual intercourse. When analyzing the entire sample, the results showed a correlation between satisfaction of body image and female genitalia. Moreover, when comparing groups with and without dyspareunia, women who experience pain while in a sexual relationship had higher levels of fear and anxiety related to their body image and more negative feelings and beliefs about their genital self-image. A study of women seeking genital aesthetic plastic surgery demonstrated an improvement in both genital image and body image after surgery. The improvement of genital image and body image was observed when the results of the participants were compared, before and within 24 months after the surgery, and after a comparison with a control group. The FGSIS was used to assess genital image, which presented an initial score of approximately 16 points. During the postoperative period, there was an improvement in genital image, and after 24 months it reached a score of approximately 24 points. However, the instrument used to evaluate body image was different from that of the present study^(23)^. As in the present study, most studies in the literature point to a relation between female body image and genital image.

Although the relation between body image and genital image as described in the present study is in agreement with the results mentioned in the literature, one study reported contradictory findings. Using a population of primiparous and sexually active women, who were on average seven months postpartum. It was observed that the dissatisfaction of body image diminished and that of the genital image increased. In addition, women who underwent vaginal delivery, unlike women who underwent a cesarean section, had higher levels of body satisfaction and a lower genital self-image^(24)^.

More recently, in a total of 69 subacute postpartum women, most participants (97%) had a positive sexual and body appreciation, with the exception of sexual pleasure, where 38% indicated they had less sexual pleasure due to genital alterations^(25)^.Therefore, in order to confirm the relation between female body and genital image, it is necessary to be aware of the different characteristics of the population. Pregnant woman in the postpartum period could experience, in addition to hormonal and psychological influences, physical changes due to pregnancy, affecting the physical perception and the relationship with their own body and/or genitalia, and consequently affecting sexual function.

In the present study, there was a significant correlation between female body and genital image, However, a weak correlation was presented in the statistical analysis^(16,19)^. Similar results were described in the surveys of DeMaria et al.,^(16,19) ^2011 and 2012, using the same questionnaire to evaluate genital image. Although studies have described the FGSIS as a reliable tool to evaluate genital image, Herbenick and Reece,^ (26)^ suggested that further research was required to understand the suitability of the FGSIS in various populations. With the results of our study, we could form the hypothesis that the FGSIS does not address the dissatisfaction of women with their genital image when dealing with esthetic factors. In Brazil, there has been an exponential increase in the search for genital plastic surgery in recent years (ISAPS, 2014), turning it into one of the most performed surgeries in the country^(14)^. Another justification for this hypothesis is based on comparisons with body image instruments with body illustrations, whereby the participant is requested to indicate the region of greatest discomfort and/or desire for change^(27).^

In the present study, 81% of the women were satisfied with their body image, differing from the data in the literature. In a review with studies in Brazilian populations, a dissatisfaction with body image in adults with scores of around 60 to 87% was shown^(2)^. Another Brazilian study identified a body image dissatisfaction rate of 85.9% for both sexes. When analyzing the characteristics of these women, the majority, approximately 60%, who were dissatisfied with the body, reported excess body weight as their main concern, even though 59% of them were classified as being “within normal range”. Moreover, the study also showed that women with a low level of physical activity were those who were most dissatisfied with being overweight^(1)^.

Underlining the findings that a high BMI has a negative influence on body image, the results of this research also showed that women with some level of concern and dissatisfactions with their body were overweight. Overweight women have more negative opinions about the perception of their weight and their bodies, and are more dissatisfied^(3,28,29,30,31,32)^. In addition to being overweight, women who were dissatisfied with their bodies were the youngest and had a worse genital image. The literature shows that dissatisfaction with body image is similar between young and older women^(33,34,35)^. Despite the similarity of satisfaction among women of different ages, younger women are more affected by influences of social imposition and media^(33,34)^.

However, the relationship between being overweight and/or age with genital image is not seen in the literature. It is possible to verify that several genital image studies have been performed in young populations at university, and that this population is satisfied with its genitalia^(16,36)^. It is believed that there is a possibility that overweight woman and woman unsatisfied with their weight, by neglecting the body, see less of their body and visualize the genitalia to be imperfect. Women who perceive their body as being overweight may have the same perception of their genitalia, and as a consequence have a justification for being dissatisfied. However, in the present study, women over 60 years were not approached for possible comparisons and the age of the women who participated in the research represented a young adult population. In this way, it is not possible to derive the relationship between body and genital image and age.

In the literature, the context of body image is addressed more often than female genital image. However, like body image, genital image is well studied in university populations^(2,7,16,37)^, and these studies barely evaluate the existence of the correlation between the body and vulva^(7,16)^. Therefore, we consider it important to study whether concerns about the body also extend to the vagina, in environments with populations with heterogeneous characteristics, and across different levels of schooling, and socio-cultural influences, even though the majority of the literature describes body image and genital image in university students. In the present study, a common demographic characteristic was that most participants completed a form of higher education, producing a similarity in the results. The similarity of body and genital image satisfaction of the present study and in the literature implies that more than the educational level of the individual, the culture and society in which one lives can influence the perception of a woman about herself. The justification of this hypothesis is based on the comparison between the financial conditions of the countries where the research was conducted. Most university studies were performed in countries with greater investment in education and culture. The present study, however, deals with women residing in a developing country.

Through questionnaires or photographs of vulvas, surveys evaluate the concepts of genitalia being considered normal and ideal, apart from the satisfaction of woman with their genitalia^(7,19)^. Due to the absence of an instrument to evaluate genital image in the Portuguese language, it is not possible to verify the main predictors associated with genital image. Moreover, the resources of evaluation of genital image recommend measuring the perception of the genitalia as a factor of satisfaction, with the absence of instruments that can quantify the impact of the appearance of the vulva through visual resources. For body image, this is already well established because there are visual instruments that allow women to point to the region that bothers them the most^(27)^. Most studies evaluate genital image with a sexual function. In this study, a correlation between body image and genital female image was shown, and questions were raised as to whether the perception of genital image interferes more with aspects of health or perception of the body.

Dissatisfaction with genital image reduces the frequency of women presenting to gynecologic offices and the amount of prevention exams^(16)^. Therefore, due to the correlation of body and genital image, health professional should, before women become dissatisfied with their body, be attentive to the vulva, and with preventive action, guide them to attend gynecologists, thereby minimizing the risk of diseases and injuries. Professionals who work in sexual health should pay attention to how women observe their body to detect dissatisfactions in genitalia. If women show eating behavior disorders due to dissatisfaction, they should be referred to more specialized professionals. Knowledge about body and genital perception may favor the behavior of health professionals in psychology, sexual and nutritional health, and could help in the recognition of possible indicators for the search for physical activity practices and esthetic procedures.

Studies that are part of the line of research of this group, seeking to analyze body and genital image in physically active and sedentary women, as well as their relationships with socio-demographic and clinical data, sexual function, and quality of life, are underway. Also, another project is the elaboration of a computational model that helps health professionals in the evaluation of female genital self-image. We suggest that future research may also evaluate the relation of men with their body and external genital organs.

### Study Limitations

From the questionnaires used, the FGSIS is not a validated instrument in the Portuguese language and with the criteria of visualization, the authors decided to make a translation because genital self-image is a relevant research topic for the Brazilian population. This limitation was minimized through reliable analysis. However, the analysis of the study refers to the reliability between the answers, but does not provide internal validity. This justifies the need for studies with a formulation of instruments for the Portuguese language because there is an increase of concerns associated with female genitalia.

## Conclusion

The dissatisfaction with body image is associated with poorer genital self-image. BMI and age are predictors of female body image perception. It was not possible to find clinical and socio-demographic data predicting female genitalia self-image factors.

## Figures and Tables

**Table 1:**
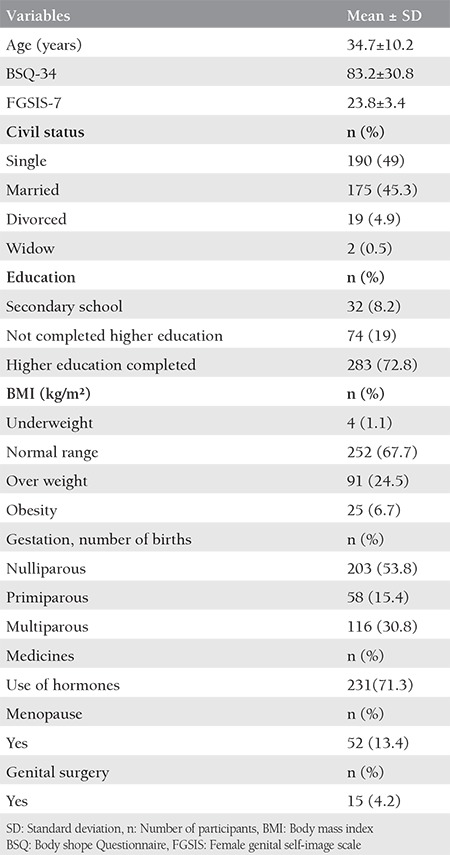
Socio-demographic and clinical characteristics of 387 women

**Table 2:**
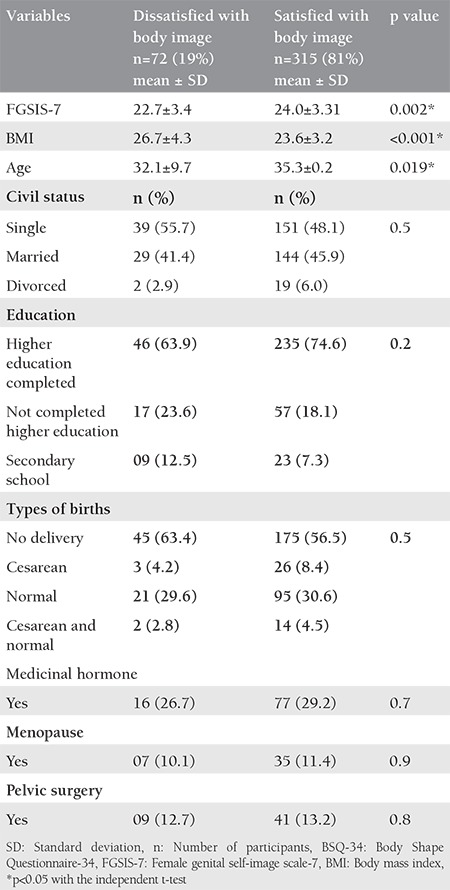
Comparison of body image (BSQ-34) with the genital image (FGSIS-7) and demographic and clinical variables of women

**Table 3:**
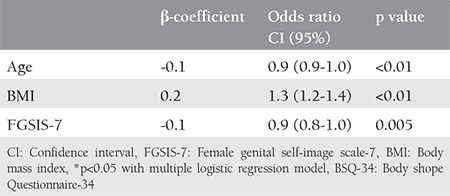
Independent variables of body satisfaction through the BSQ-34
